# Lack of the Transient Receptor Potential Vanilloid 1 Shifts Cannabinoid-Dependent Excitatory Synaptic Plasticity in the Dentate Gyrus of the Mouse Brain Hippocampus

**DOI:** 10.3389/fnana.2021.701573

**Published:** 2021-07-07

**Authors:** Jon Egaña-Huguet, Miquel Saumell-Esnaola, Svein Achicallende, Edgar Soria-Gomez, Itziar Bonilla-Del Río, Gontzal García del Caño, Sergio Barrondo, Joan Sallés, Inmaculada Gerrikagoitia, Nagore Puente, Izaskun Elezgarai, Pedro Grandes

**Affiliations:** ^1^Department of Neurosciences, Faculty of Medicine and Nursing, University of the Basque Country UPV/EHU, Leioa, Spain; ^2^Achucarro Basque Center for Neuroscience, Science Park of the University of the Basque Country UPV/EHU, Leioa, Spain; ^3^Department of Pharmacology, Faculty of Pharmacy, Centro de Investigación Biomédica en Red de Salud Mental, University of the Basque Country UPV/EHU, Vitoria-Gasteiz, Spain; ^4^Bioaraba, Neurofarmacología Celular y Molecular, Vitoria-Gasteiz, Spain; ^5^IKERBASQUE, Basque Foundation for Science, Bilbao, Spain; ^6^Department of Neurosciences, Faculty of Pharmacy, University of the Basque Country UPV/EHU, Vitoria-Gasteiz, Spain; ^7^Division of Medical Sciences, University of Victoria, Victoria, BC, Canada

**Keywords:** endovanilloid system, CB_1_ receptor, excitatory synapses, long-term potentiation, G proteins

## Abstract

The transient receptor potential vanilloid 1 (TRPV1) participates in synaptic functions in the brain. In the dentate gyrus, post-synaptic TRPV1 in the granule cell (GC) dendritic spines mediates a type of long-term depression (LTD) of the excitatory medial perforant path (MPP) synapses independent of pre-synaptic cannabinoid CB_1_ receptors. As CB_1_ receptors also mediate LTD at these synapses, both CB_1_ and TRPV1 might be influencing the activity of each other acting from opposite synaptic sites. We tested this hypothesis in the MPP–GC synapses of mice lacking TRPV1 (TRPV1-/-). Unlike wild-type (WT) mice, low-frequency stimulation (10 min at 10 Hz) of TRPV1-/- MPP fibers elicited a form of long-term potentiation (LTP) that was dependent on (1) CB_1_ receptors, (2) the endocannabinoid 2-arachidonoylglycerol (2-AG), (3) rearrangement of actin filaments, and (4) nitric oxide signaling. These functional changes were associated with an increase in the maximum binding efficacy of guanosine-5′-O-(3-[^35^S]thiotriphosphate) ([^35^S]GTPγS) stimulated by the CB_1_ receptor agonist CP 55,940, and a significant decrease in receptor basal activation in the TRPV1-/- hippocampus. Finally, TRPV1-/- hippocampal synaptosomes showed an augmented level of the guanine nucleotide-binding (G) Gα_i1_, Gα_i2_, and Gα_i3_ protein alpha subunits. Altogether, the lack of TRPV1 modifies CB_1_ receptor signaling in the dentate gyrus and causes the shift from CB_1_ receptor-mediated LTD to LTP at the MPP–GC synapses.

## Introduction

Cannabinoid functions in the brain are typically associated with the activation of cannabinoid CB_1_ receptors (Kano et al., 2009; Katona and Freund, 2012; Pertwee, 2015; Lu and Mackie, 2016). Additionally, endogenous, plant-derived, and synthetic cannabinoids could have other molecular targets. In particular, several cannabinoid effects depend on the activation of members of the transient receptor potential (TRP) channel family (De Petrocellis et al., 2011; Morales and Reggio, 2017; Muller et al., 2019). For example, the synthetic cannabinoid WIN 55,212-2 exerts analgesic effects by acting on the transient receptor potential vanilloid 1 (TRPV1) (Jeske et al., 2006; Patwardhan et al., 2006; Ruparel et al., 2011).

The TRPV1 receptors are expressed in different brain regions and cell types (Tóth et al., 2005; Cristino et al., 2006; Cavanaugh et al., 2011). Particularly in the hippocampus, functional TRPV1 receptors in the long term depress excitatory pyramidal cell–interneuron synapses (Gibson et al., 2008), and pre-synaptic TRPV1 facilitates the release of glutamate at the excitatory synaptic terminals in the CA1 hippocampus (Bialecki et al., 2020). TRPV1 also localizes to the post-synaptic sites of excitatory and inhibitory synaptic terminals in the molecular layer (ML) of the dentate gyrus where it plays a key role in the synaptic transmission and plasticity (Chávez et al., 2010; Chavez et al., 2014; Canduela et al., 2015; Puente et al., 2015). Thereby, like in other brain regions (Lafourcade et al., 2007; Grueter et al., 2010; Puente et al., 2011), long-term depression (LTD) at medial perforant path (MPP) synapses is mediated by post-synaptic TRPV1 activity (Chávez et al., 2010) and CB_1_ receptor signaling (Peñasco et al., 2019; Fontaine et al., 2020). Conversely, high-frequency stimulation (HFS) of the lateral perforant path (LPP) triggers a CB_1_ receptor-dependent long-term potentiation (LTP) that requires post-synaptic *N*-methyl-_D_-aspartate (NMDA) receptors, metabotropic glutamate receptor 5 (mGluR5), and mobilization of the endocannabinoid 2-arachidonoylglycerol (2-AG) (Wang et al., 2016). Anatomically, the constitutive absence of TRPV1 in mice alters the principal degrading enzymes of 2-AG and anandamide (AEA, the other main endocannabinoid), as well as modifies CB_1_ receptors localized to the excitatory and inhibitory terminals in the outer two-third of ML, the termination zone of the perforant path (Egaña-Huguet et al., 2021). However, the functional impact of these adaptive changes on synaptic plasticity is currently unknown. In this study, we addressed this question by investigating the effects of the constitutive lack of TRPV1 on the CB_1_ receptor-dependent LTD of the excitatory MPP synapses.

## Materials and Methods

### Animals

All protocols were approved by the Committee of Ethics for Animal Welfare of the University of the Basque Country (CEEA/M20/2015/105; CEIAB/M30/2015/106) and were in accordance with the European Communities Council Directive of September 22, 2010 (2010/63/EU) and Spanish regulations (Real Decreto 53/2013, BOE 08-02-2013). All efforts were made to minimize pain and suffering and to reduce the number of animals used. Seven-/eight-week-old male TRPV1-/- mice (*n* = 43) and their WT littermates (TRPV1+/+) (*n* = 21) were used. The TRPV1-/- mice were derived from heterozygous breeding pairs as described previously in the study by Egaña-Huguet et al. (2021). Mice were housed in pairs or groups of maximum three littermates in standard Plexiglas cages (17 ×14.3 ×36.3 cm), and before the experiments were conducted, they were allowed to acclimate to the environment for at least 1 week. They were maintained at standard conditions with food and tap water *ad libitum* throughout all experiments and in a room with a constant temperature (22°C), and kept in a 12:12 h light/dark cycle with lights off at 9:00 p.m.

### Slice Preparation for Electrophysiology

TRPV1-/- and WT mice were anesthetized by the inhalation of isoflurane. After decapitation, their brains were rapidly removed and placed on ice-cold sucrose-based solution that contained the following components (in mM): 87 NaCl, 75 sucrose, 25 glucose, 7 MgCl_2_, 2.5 KCl, 0.5 CaCl_2_, and 1.25 NaH_2_PO_4_. Coronal sections (300 μm thick) were cut with a vibratome (Leica Microsystems S.L.U.), then were recovered at 32–35°C, and were superfused (2 mL/min) in the recording chamber with artificial cerebrospinal fluid (ACSF) containing the following components (in mM): 130 NaCl, 11 glucose, 1.2 MgCl_2_, 2.5 KCl, 2.4 CaCl_2_, 1.2 NaH_2_PO_4_, and 23 NaHCO_3_, equilibrated with 95% O_2_/5% CO_2_. All experiments were carried out at 32–35°C. The superfusion medium contained picrotoxin (PTX) (100 μM). All drugs were added to their final concentration in the superfusion medium. For the extracellular field recordings, a glass recording pipette was filled with ACSF. The stimulation electrode was placed in the MPP (middle one-third of the ML) or LPP (outer one-third of the ML), and the recording pipette was always placed in the inner one-third of the dentate ML (mossy cell fiber layer).

A low-frequency stimulation (LFS, 10 min at 10 Hz) was applied to induce endocannabinoid-dependent excitatory LTD (eCB-eLTD) of glutamatergic inputs following the recording of a steady baseline in the presence of drugs (Puente et al., 2011; Peñasco et al., 2019). The fEPSP slope, area, and amplitude were measured (graphs depict the area). MPP stimulation was confirmed by the group II mGluRs agonist LY354740. Consistent with previous reports (Macek et al., 1996; Chiu and Castillo, 2008; Chávez et al., 2010), 1 μM of LY354740 strongly reduced MPP-fEPSPs by 59.60 ± 1.451% 10 min after the drug application (*n* = 4, ^**^p < 0.002) (data not shown). The magnitude of the eCB-eLTD after the LFS stimulation was calculated as the percentage change between baseline (averaged excitatory responses for 10 min before LFS) and last 10 min of stable responses, normally at 30 min after the end of the LFS. The slices used for each experimental condition (*n*) were obtained from at least three mice.

### Extracellular Field Recordings

To evoke field excitatory post-synaptic potential responses (fEPSPs), repetitive control stimuli were delivered at 0.1 Hz (Stimulus Isolator ISU 165, Cibertec, Spain; controlled by a Master-8, A.M.P.I.). An Axopatch-200B (Axon Instruments/Molecular Devices, Union City, CA, United States) was used to record the data filtered at 1–2 kHz, digitized at 5 kHz on a DigiData 1440A interface (Axon Instruments/Molecular Devices, Union City, CA, United States). Data were collected on a PC using Clampex 10.0 (Axon Instruments/Molecular Devices, Union City, CA, United States) and analyzed using Clampfit 10.0 (Axon Instruments/Molecular Devices, Union City, CA, United States). At the start of each experiment, an input–output curve was constructed. A stimulation intensity was selected for baseline measurements that yielded between 40 and 60% of the maximal amplitude response.

### Hippocampal Membrane Preparation

Synaptosomes were prepared as previously described by the study by Garro et al. (2001). TRPV1-/- and WT mice (*n* = 7 each) were anesthetized with isoflurane and decapitated; the brains were removed and placed on ice-cold 0.32 M sucrose, pH 7.4, containing 80 and 20 mM NaH_2_PO_4_ (sucrose phosphate buffer) with protease inhibitors (iodoacetamide 50 μM, PMSF 1 mM). The hippocampal tissue was minced and homogenized in 10 volumes of sucrose/phosphate buffer using a motor-driven Potter Teflon glass homogenizer (motor speed 800 rpm; 10 up and down strokes; mortar cooled in an ice-water mixture throughout). The homogenate was centrifuged at 1,000 × *g* for 10 min, and the obtained pellet (P1) was re-suspended and pelleted. The supernatants (S1 + S1′) were pelleted at 15,000 × g (P2) and re-suspended in the homogenization buffer to a final volume of 16 mL. The suspension was layered directly onto the tubes containing 8 ml of 1.2 M sucrose/phosphate buffer and centrifuged at 180,000 × *g* for 20 min. The material retained at the gradient interface was carefully collected with a Pasteur pipette and diluted with the ice-cold 0.32 M sucrose/phosphate buffer to a final volume of 16 ml. The diluted suspension was then layered onto 8 ml of 0.8 M sucrose/phosphate buffer and centrifuged as described above. The obtained pellet was re-suspended in the ice-cold phosphate buffer, pH 7.5, and aliquoted in microcentrifuge tubes. Aliquots were then centrifuged at 40,000 × *g* for 30 min, the supernatants were aspirated, and the pellets corresponding to the nerve terminal membranes were stored at −80°C. The protein content was determined using the Bio-Rad dye reagent with bovine γ-globulin as a standard.

### Western Blotting of Hippocampal Synaptosomes

Hippocampal extracts from TRPV1-/- and WT were boiled in urea-denaturing buffer [20 mM Tris–HCl, pH 8.0, 12% glycerol, 12% urea, 5% dithiothreitol, 2% sodium dodecyl sulfate (SDS), and 0.01% bromophenol blue] for 5 min. Increasing concentrations (2, 4, 8, 12, 16, and 20 μg/μL) of denatured proteins were resolved by electrophoresis on SDS–polyacrylamide (SDS–PAGE) gels (10%) using the Mini Protean II gel apparatus (Bio-Rad, Hercules, CA, United States). Proteins were transferred to polyvinylidene fluoride (PVDF) membranes (Amersham Biosciences, Buckinghamshire, United Kingdom) using the Mini TransBlot transfer unit (Bio-Rad, Hercules, CA, United States) at 90 V constant voltage for 1 h at 4°C. Blots were blocked in 5% non-fat dry milk/PBS containing 0.5% BSA and 0.2% Tween for 1 h, and incubated overnight at 4°C with anti-Gαo (0.04 ng/μL: Santa Cruz Biotechnology; rabbit polyclonal: K-20; AB_2314438), anti-Gαi_1_ (0.2 ng/μL; Santa Cruz Biotechnology; rabbit polyclonal: sc-391, AB_2247692), anti-Gαi_2_ (0.2 ng/μL; Santa Cruz Biotechnology; rabbit polyclonal: sc-7276, AB_2111472), and anti-Gαi_3_ antibodies (4 pg/μl; Santa Cruz Biotechnology; rabbit polyclonal: sc-262, AB_2279066). Blots were washed and incubated with horseradish peroxidase (HRP)-conjugated secondary antibody goat anti-rabbit IgG HRP (1 ng/mL; Cell Signalling Technology; 7074; RRID: AB_2099233) diluted to 1:10,000 in the blocking buffer for 2 h at room temperature. Immunoreactive bands were incubated with the ECL system according to the manufacturer's instructions and detected in an Autochemi-UVP Bioimaging System. Then, the bands were quantified with Image-J (FIJI) (NIH, United States; RRID: SCR_003070), and the differences between the relative expressions of proteins were analyzed by the regression line slope comparison method using a statistical software package (see Supplementary Material). The protein loading was determined by the Coomassie Brilliant Blue gel staining method (see Supplementary Material) (GraphPad Prism, GraphPad Software Inc, San Diego, United States).

### [^35^S]GTPγS Binding Assays

Hippocampal extracts (25 μg protein) were thawed and incubated at 30°C for 2 h in [^35^S]GTPγS-incubation buffer (0.5 nM [^35^S]GTPγS, 1 mM EGTA, 3 mM MgCl_2_, 100 mM NaCl, 0.2 mM DTT, 50 μM GDP, and 50 mM Tris–HCl, pH 7.4). The CB_1_ receptor agonist CP 55,940 (10^−11^-10^−5^ M, eight concentrations) was added to determine the receptor-stimulated [^35^S]GTPγS binding. Non-specific binding was defined in the presence of 10 μM unlabeled GTPγS. Basal binding was assumed to be the specific [^35^S]GTPγS binding in the absence of an agonist. The reactions were terminated by the rapid vacuum and filtration through Whatman GF/C glass fiber filters, and the remaining bound radioactivity was measured by the liquid scintillation spectrophotometry. For the analysis of data, individual CP 55,940 concentration–response curves were fitted by the non-linear regression method to the four-parameter Hill equation, using GraphPad Prism (GraphPad Prism, GraphPad Software Inc, San Diego, United States):

$$\begin{array}{l} \text{E}=\text{Basal}+{\text{E}}_{\text{max}}-Basal/1+10\left(LogEC_{50}-Log\left[A\right]\right)nH \end{array}$$

where E denotes the effect, log [A] the logarithm of the concentration of agonist, nH the midpoint slope, LogEC_50_ the logarithm of the midpoint location parameter, and E_max_ and Basal the upper and lower asymptotes, respectively. When required, simultaneous model fitting with parameter sharing across the datasets was performed using GraphPad Prism (GraphPad Prism, GraphPad Software Inc, San Diego, United States). The EC_50_ values were transformed into pEC_50_ (–LogEC_50_) as EC_50_, and affinity constants obtained experimentally are log-normally distributed. Therefore, statistical analysis was performed accordingly (Christopoulos, 1998).

### Drugs and Chemicals

All drugs for performing electrophysiological studies were dissolved in dimethyl sulfoxide (DMSO; Sigma-Aldrich) and were added at the final concentration to the superfusion medium. CP 55,940, AMG9810, LY354740, AM251, URB597, JZL 184, tetrahydrolipstatin (THL), latrunculin A (LAT-A), and PTX were purchased from Tocris BioScience (Bristol, United Kingdom). *S*-Nitroso-*N*-acetylpenicillamine (SNAP) was purchased from Abcam (Cambridge, MA). All drugs were perfused at least 20 min before the LFS protocol apart from JZL 184. JZL 184 was preincubated at least 1 h before LFS protocol.

### Experimental Design and Statistical Analysis

All values are given as mean ± SEM with *p*-values and sample size (*n*). Shapiro–Wilk and Kolmogorov–Smirnov tests were used to confirm the normality of the data. Statistical significance between groups was tested using parametric or non-parametric two-tailed Student's *t*-test as required. The significance level was set at *p* < 0.05 for all comparisons. All statistical tests were performed with GraphPad Prism (GraphPad Prism, GraphPad Software Inc, San Diego, United States).

## Results

### Synaptic Potentiation of the MPP Synapses in TRPV1-/- Mice

**Figure 1:**
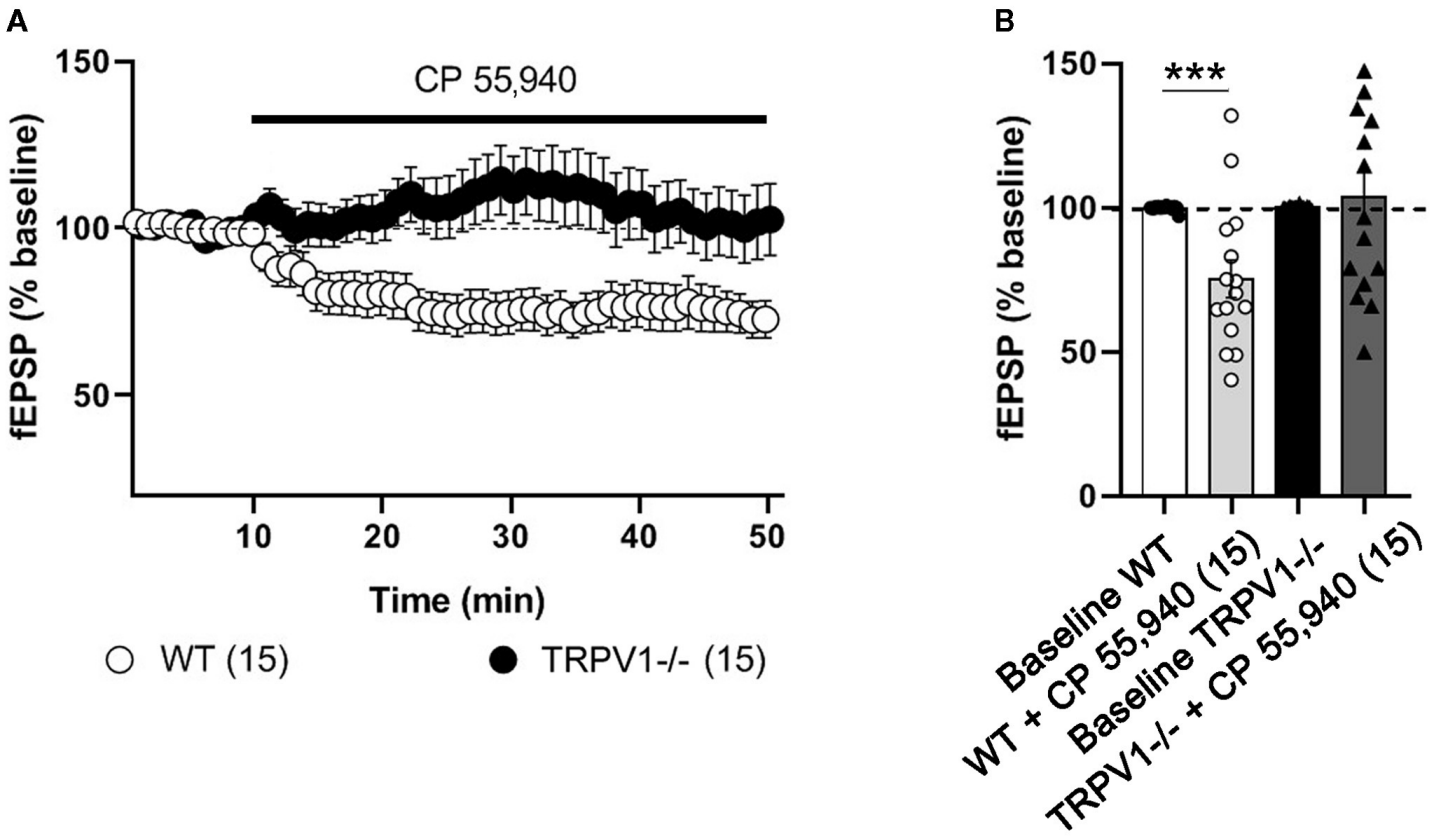
Excitatory synaptic transmission at the medial perforant path (MPP) synapses in WT and TRPV1-/- mice. For representation, experiments were normalized to its baseline. **(A)** Time-course plot of average fEPSP areas is represented. Black arrow indicates the time point when the drug was applied. CP 55,940 [10 μM] reduces significantly the excitatory synaptic transmission in WT, but not in TRPV1-/-. **(B)** Representative histograms of the last 10 min of fEPSP after CP 55,940 application in WT (Mann–Whitney test. ***p < 0.001) and TRPV1-/- (Mann–Whitney test. *p* > 0.05) mice. All data are expressed as mean ± SEM.

As expected in WT littermates (Peñasco et al., 2019), fEPSPs were significantly reduced after applying the CB_1_ receptor agonist CP 55,940 [(10 μM); 75.31 ± 6.496%; ^***^p = 0.0003 vs. baseline] (Figures 1A,B). In contrast, CP 55,940 did not change fEPSPs in TRPV1-/- mice [(10 μM); 103 ± 8.978%; ns; *p* = 0.7748 vs. baseline] (Figures 1A,B).

**Figure 2:**
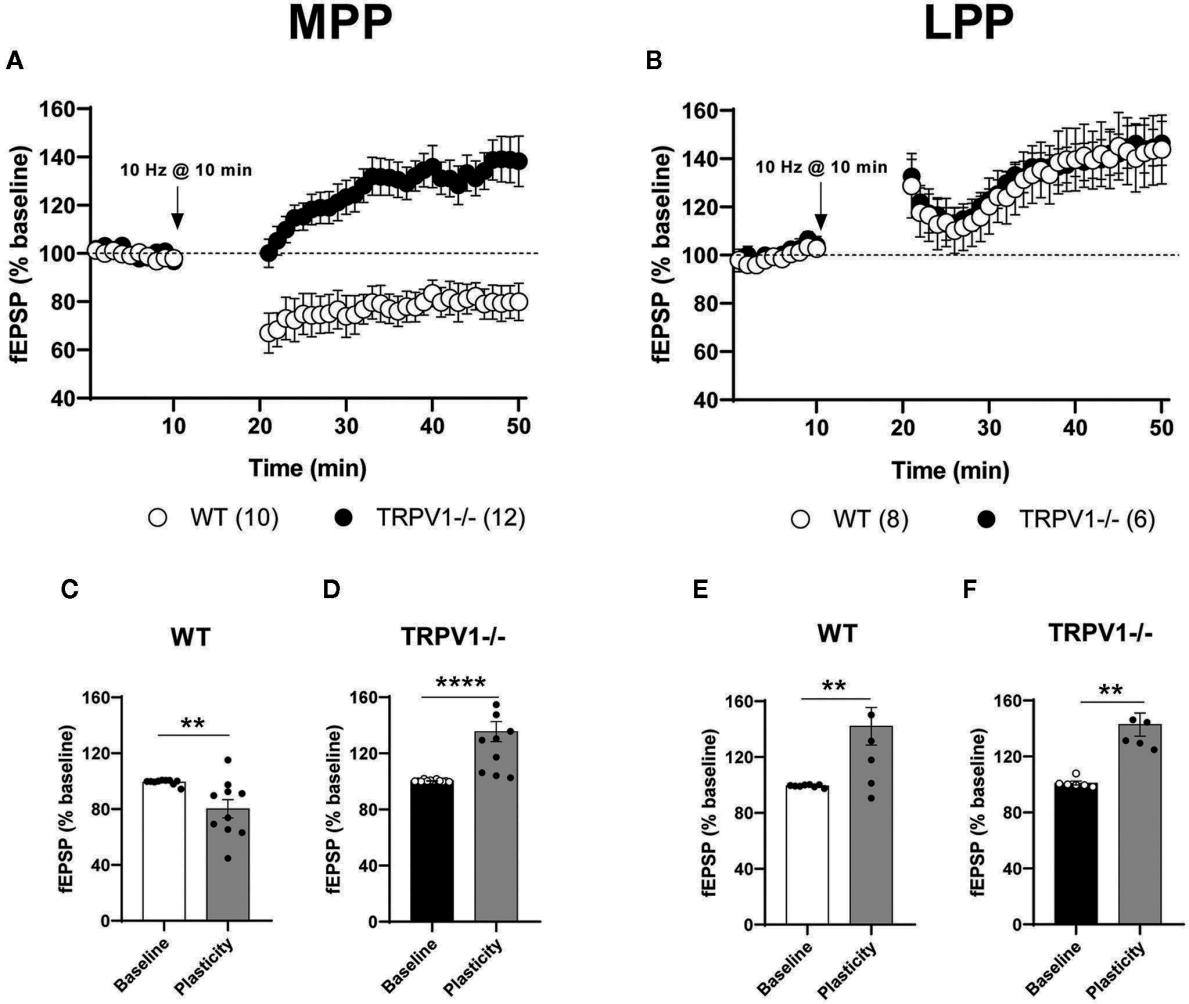
**(A)** Low-frequency stimulation (LFS) triggers long-term depression (LTD) at the medial perforant path (MPP) synapses in WT (white circles) and long-term potentiation (LTP) in TRPV1-/- (black circles). **(B)** Similar stimulation elicits the long-term potentiation at the lateral perforant path (LPP) in WT (white circles) and TRPV1-/- (black circles). For representation, each experiment was normalized to its baseline. The average of the fEPSP areas is shown. Arrows point to LFS application (10 min, 10 Hz). Representative histograms of fEPSP (last 10 min) after LFS at MPP in: **(C)** WT (Mann–Whitney test. **p < 0.005 vs. baseline), **(D)** TRPV1-/- (Mann–Whitney test. ****p < 0.0001 vs. baseline), and after LFS at LPP in **(E)** WT (unpaired t-test. **p < 0.005 vs. baseline), and **(F)** TRPV1-/- (Mann–Whitney test. **p < 0.005 vs. baseline). All data are expressed as mean ± SEM.

**Figure 3:**
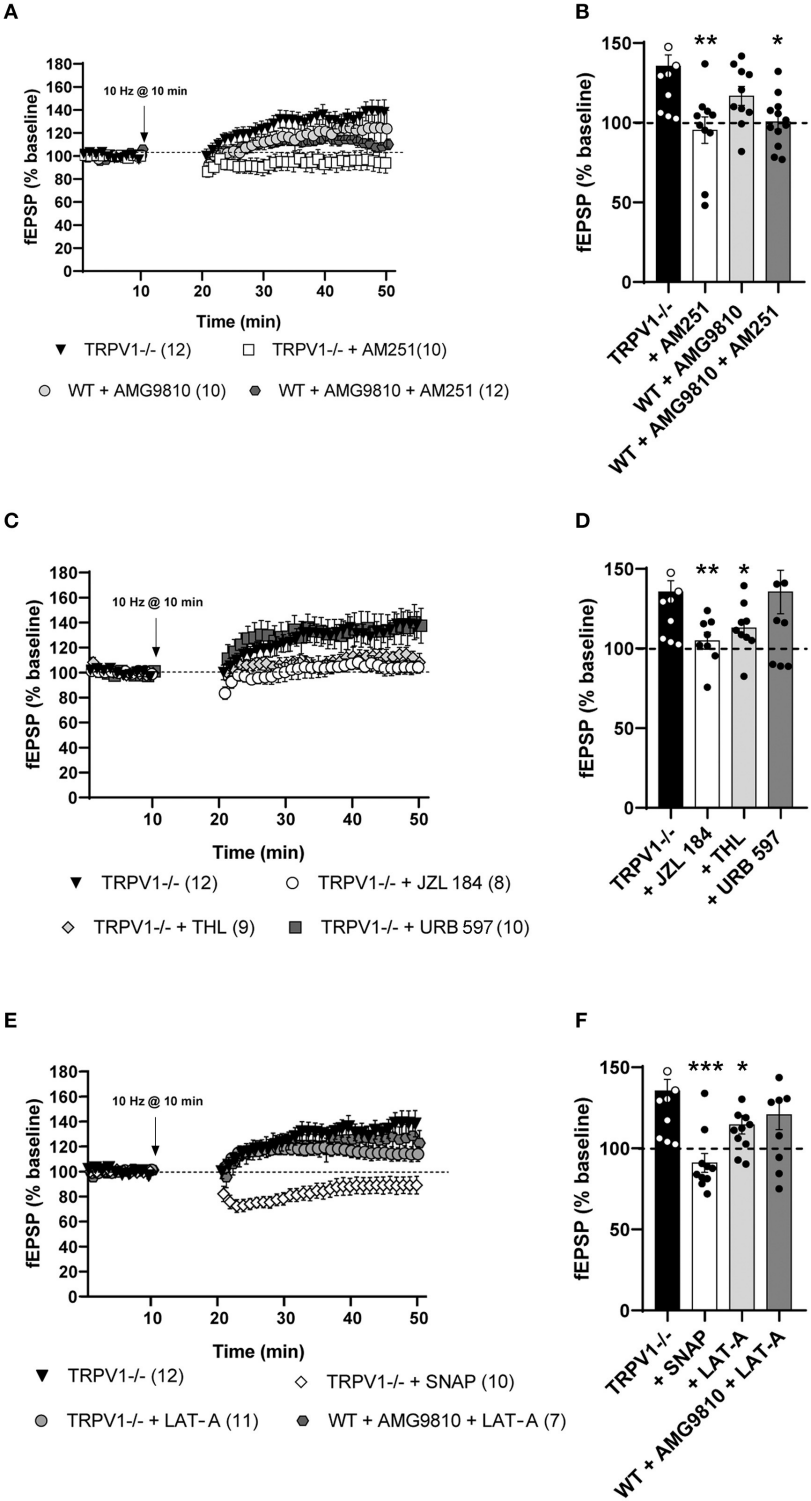
**(A)** Medial perforant path long-term potentiation (MPP-LTP) in TRPV1-/- is CB_1_ receptor-dependent (Mann–Whitney test. *p* > 0.05 vs. baseline; white squares). The TRPV1 antagonist AMG9810 (3 μM) triggers MPP-LTP in WT (Mann–Whitney test. *p < 0.05 vs. baseline; light gray circles) that is also CB_1_ receptor-dependent (Mann–Whitney test. *p* > 0.05 vs. baseline; dark gray hexagons). **(B)** Representative histograms of fEPSP (last 10 min) after LFS at MPP in different conditions: TRPV1-/- mice (black bar); TRPV1-/- + AM251 [4 μM] (white bar); WT + AMG9810 [3 μM] (light gray bar); and WT + AMG9810 [3 μM] + AM251 [4 μM] (dark gray bar). Statistical analysis of last 10 min of fEPSP after LFS in the MPP from different conditions: TRPV1-/- vs. TRPV1-/- + AM251 [4 μM] (Mann–Whitney test. **p < 0.005); TRPV1-/- vs. WT + AMG9810 [3 μM] (Mann–Whitney test. *p* > 0.05) and WT + AMG9810 vs. WT + AMG9810 [3 μM] + AM251 [4 μM] mice (unpaired *t-*test. *p < 0.05). **(C)** Increase (Mann–Whitney test. *p* > 0.05 vs. baseline; white circles) and decrease (Mann–Whitney test. **p < 0.005 vs. baseline; light gray diamonds) in 2-AG abolish MPP-LTP in TRPV1-/-, while AEA increase (Mann–Whitney test. *p* > 0.05 vs. baseline; dark gray squares) has no effect. **(D)** Representative histogram of fEPSP (last 10 min) after LFS at MPP in TRPV1-/- (black bar) in the presence of JZL 184 [50 μM; >1 h] (white bar), THL [10 μM] (light gray bar), and URB597 [2 μM] (dark gray bar). Statistical analysis of last 10 mins fEPSP after LFS in the MPP of TRPV1-/- mice in the presence of different drugs: TRPV1-/- vs. TRPV1-/- + JZL 184 (unpaired *t*-test. **p < 0.005), TRPV1-/- vs. TRPV1-/- + THL (unpaired *t*-test. *p < 0.05), and TRPV1-/- vs. TRPV1-/- + URB597 (unpaired *t*-test. *p* > 0.05). **(E)** SNAP blocks (Mann–Whitney test. *p* > 0.05 vs. baseline; white diamonds) and LAT-A reduce MPP-LTP in TRPV1-/- (Mann–Whitney test. *p < 0.05 vs. baseline; light gray circles) but not LTP induced by AMG9810 in WT (Mann–Whitney test. *p* > 0.05 vs. baseline; dark gray hexagons). **(F)** Representative histogram of the last 10 min of fEPSP after LFS in different conditions: SNAP in TRPV1-/- [100 μM] (white bar); LAT-A in TRPV1-/- (light gray bar), and LAT-A in WT + AMG9810 mice (dark gray bar). Statistical analysis of last 10 min of fEPSP after LFS in the MPP of different conditions: TRPV1-/- vs. TRPV1-/- + SNAP (Mann–Whitney test. ***p < 0.001); TRPV1-/- vs. TRPV1-/- + LAT-A (unpaired *t-*test. *p < 0.05); and WT + AMG9810 vs. WT + AMG9810 + LAT-A (unpaired *t*-test. *p* > 0.05). All data are expressed as mean ± SEM. **(A,C,E)** For representation, each experiment was normalized to its baseline. The average of the fEPSP areas is shown. Arrows point to LFS application (10 min, 10 Hz).

The fEPSPs were significantly reduced after low LFS (10 min at 10 Hz) of MPP in WT littermates (80.21 ± 6.497%; ^**^p = 0.0021 vs. baseline) (Figures 2A,C). However, a significant fEPSP potentiation (MPP-LTP) was observed in TRPV1-/- mice after the similar stimulation pattern (135 ± 7.119%; ^****^p < 0.0001 vs. baseline) (Figures 2A,D). This LTP was significantly reversed by the CB_1_ receptor antagonist AM251, indicating that CB_1_ receptors were involved [(4 μM); 95.37 ± 8.263%; ns; *p* > 0.9999 vs. baseline; ^**^p = 0.0034 vs. the fEPSP potentiation obtained in TRPV1-/-] (Figures 3A,B, respectively). Moreover, bath application of the TRPV1 antagonist AMG9810 significantly potentiated fEPSPs at the MPP synapses of WT [(3 μM); 116.8 ± 5.957%; ^*^p = 0.0185 vs. baseline] (Figure 3A); however, it was not as robust as in TRPV1-/- [(3 μM): 116.8 ± 5.957%; ns; *p* = 0.0638 vs. potentiation value from TRPV1-/-] (Figure 3B). In addition, the potentiation obtained by blocking TRPV1 receptors was also reduced by AM251 [(4 μM); 100.6 ± 4.653%; ns; *p* = 0.9656 vs. baseline] (Figure 3A) to a similar extent to the reduction caused by AM251 in TRPV1-/- [WT mice with AMG9810 (3 μM) + AM251 (4 μM): 100.6 ± 4.653%; ^*^p = 0.0413 vs. the potentiation value from WT + AMG9810] (Figure 3B). Together, these data suggest that TRPV1 blockade can shift the CB_1_ receptor-dependent LTD to LTP elicited by LFS of MPP. In contrast to the results obtained in the MPP, the application of the 10 Hz stimulation at LPP synapses did not induce a depression in the WT littermates; rather, it produced a significant potentiation (142 ± 13.44 %: ^**^p = 0.0068 vs. baseline) (Figures 2B,E), as previously described in the study by Wang et al. (2016). This LPP-LTP was maintained in the LPP synapses of TRPV1-/- mice (142.9 ± 8.314 %; ^**^p = 0.0022 vs. baseline) (Figures 2B,F).

### Endocannabinoid-Mediated Synaptic Potentiation of the MPP Synapses in TRPV1-/- Mice

The MPP-LTP in TRPV1-/- mice was blocked by 1-h pre-incubation with the monoacylglycerol lipase (MAGL) inhibitor JZL 184 [(50 μM); 104.9 ± 5.317%; ns; *p* = 0.1304 vs. baseline; ^**^p = 0.0058 vs. the potentiation value from TRPV1-/-] (Figures 3C,D, respectively) and significantly reduced by THL, a diacylglycerol lipase (DAGL) inhibitor [(10 μM); 112.9 ± 5.295%; ^**^p = 0.0040 vs. baseline; ^*^p = 0.0273 vs. the potentiation value from TRPV1-/-] (Figures 3C,D, respectively). However, the fatty acid amide hydrolase (FAAH) inhibitor URB597 was ineffective at inhibiting MPP-LTP in TRPV1-/- [(2 μM); 135.5 ± 13.65%; ns; *p* = 0.1431 vs. baseline; ns; *p* = 0.9989 vs. the potentiation value from TRPV1-/-] (Figures 3C,D, respectively). These data suggest that CB_1_ receptors and 2-AG (but no AEA) are required for MPP-LTP in TRPV1-/- mice. Furthermore, the nitric oxide (NO) donor SNAP blocked MPP-LTP in TRPV1-/- [(0.1 mM); 91.02 ± 5.804%; ^*^p = 0.0232 vs. baseline; ^***^p = 0.0003 vs. potentiation value from TRPV1-/-] (Figures 3E,F, respectively).

Polymerization of actin filaments was shown to be necessary for the LTP driven by CB_1_ receptors in the LPP synapses (Wang et al., 2016, 2018). In TRPV1-/- mice, the actin polymerization inhibitor LAT-A significantly reduced MPP-LTP [(0.5 mM); 114.6 ± 5.78%; ^*^p = 0.0104 vs. baseline; ^*^p = 0.0348 vs. potentiation value from TRPV1-/-] (Figures 3E,F, respectively). LAT-A, in turn, did not modify the LTP elicited by AMG9810 in WT (120.9 ± 9.248 %; ns; *p* = 0.1431 vs. baseline; ns; *p* = 0.2173 vs. potentiation value from TRPV1-/-) (Figures 3E,F, respectively).

### Expression of Gαi/o Subunits and CP 55,940 Stimulated [^35^S]GTPγS Binding in Synaptosomal Fractions From TRPV1-/- and WT Hippocampi

**Figure 4:**
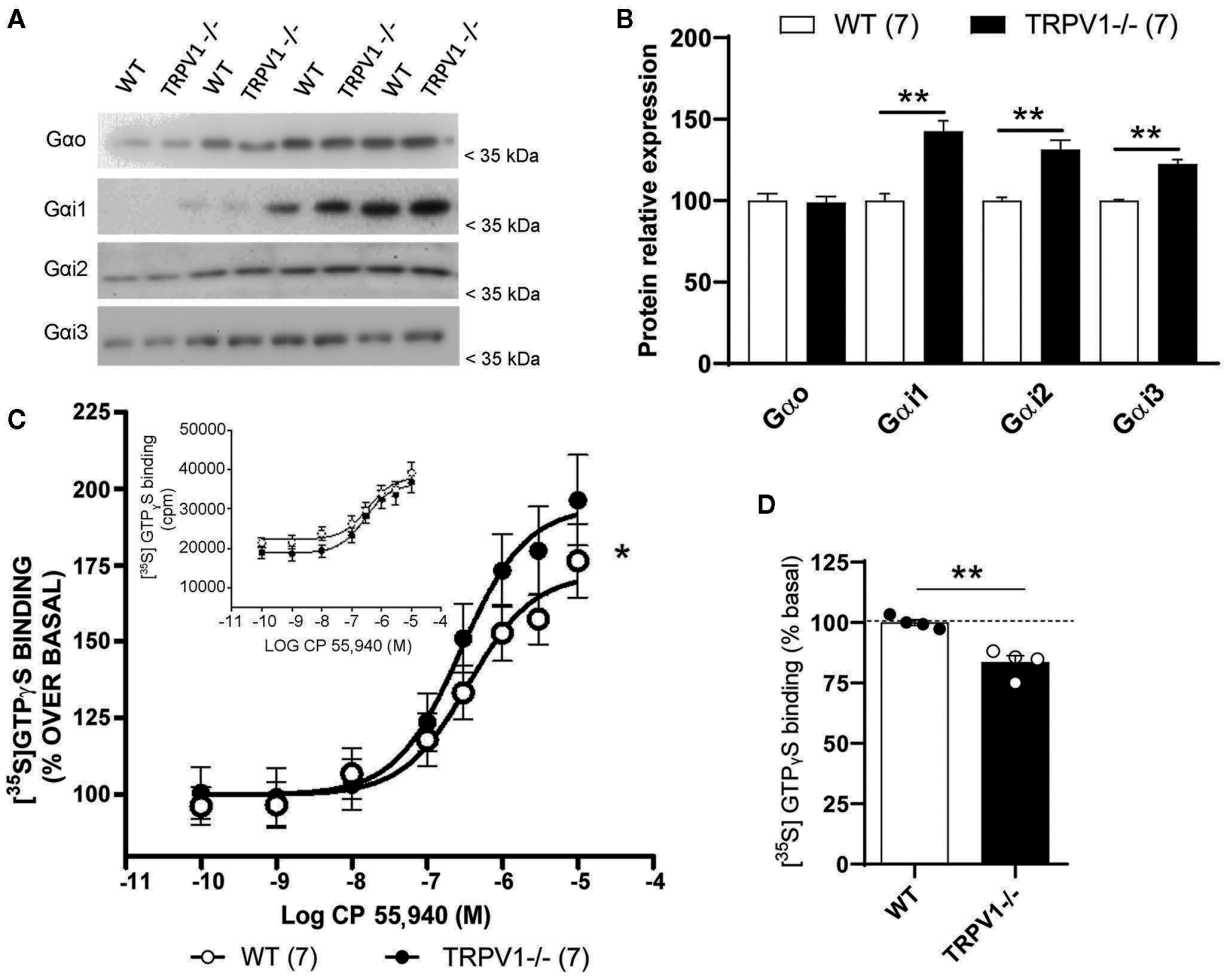
Molecular changes in Gα_o_ and Gα_i_ subunits in TRPV1-/-. **(A,B)** Immunoblot and relative expression of Gα_o_, Gα_i1_, Gα_i2_, and Gα_i3_ proteins in synaptosomal extracts with increasing protein concentrations (4, 8, 12, and 16 μg/μl) from WT and TRPV1-/- mice hippocampi. *p* > 0.05; *p < 0.05; **p < 0.005. Data are expressed as mean ± SEM. **(C)** CP 55,940-stimulated [^35^S]GTPγS binding assay in the hippocampal synaptosome fractions from WT and TRPV1-/-. Concentration curves were constructed using mean values ± SEM from four different experiments performed in triplicate. Paired *t*-test; *p < 0.05. Inset: CP 55,940 concentration–response curves are expressed in cpm (counts per minute) to show non-normalized basal (WT: 22.179 ± 2.844 cpm vs. TRPV1-/-: 18.715 ± 2.816. Paired *t*-test; *p < 0.05) and E_max_ values (WT: 39.079 ± 2.844 cpm vs. TRPV1-/-: 36.251 ± 2.285 cpm. Paired *t*-test; ns; *p* > 0.05). **(D)** Bar graph representing the relative percentage of [^35^S]GTPγS basal binding levels in WT and TRPV1-/-. Unpaired *t*-test; **p < 0.005. Data are represented as mean ± SEM.

There were no significant differences in the Gα_o_ subunit expressed in the synaptosomal fractions of TRPV1-/- and WT (TRPV1-/-: 6.25 ± 0.1598 vs. WT: 6.32 ± 0.1582; ns; *p* = 0.8633) (Figures 4A,B). However, Gα_i1_ (TRPV1-/-: 11.20 ± 0.0892 vs. WT: 7.85 ± 0.1273; ^**^p = 0.0056), Gα_i2_ (TRPV1-/-: 8.399 ± 0.1191 vs. WT: 6.399 ± 0.1563; ^**^p = 0.0065), and Gα_i3_ subunits (TRPV1-/-: 8.142 ± 0.1228 vs. WT: 6.646 ± 0.1505; ^**^p = 0.015) (Figure 4B) were significantly increased in TRPV1-/- mouse hippocampus.

The CB_1_ receptor agonist CP 55,940 stimulated [^35^S]GTPγS binding in a concentration-dependent manner (Figure 4C). The maximum efficacy (E_max_) significantly increased in TRPV1-/- (193.70 ± 12.21% of the basal activation) relative to WT (maximum efficacy: 176.20 ± 9.73% of the basal activation; ^*^p < 0.05) (Figure 4C), with no changes in potency (pEC_50_ in TRPV1-/-: 6.58 ± 0.01 vs. WT: 6.47 ± 0.14; ns; *p* = 0.5139) (Figure 4C). Also, a significant decrease in the basal activation of the receptor was detected (TRPV1-/-: 83.45 ± 2.88% vs. WT: 100 ± 1.24%; ^**^p = 0.0019) (Figure 4D). Agonist's maximal efficacy, which is defined as the maximal difference between specific [35S]GTPyS binding in the presence and absence of agonist, was calculated as the percentage of basal activation. However, if the data were not normalized to their corresponding basal values, Emax differences were not statistically significant (see captions and the inset of Figure 4C).

## Discussion

The aim of this investigation was to study the impact of the genetic deletion of TRPV1 on the CB_1_ receptor functionality and synaptic plasticity in the hippocampal dentate gyrus. Particularly, the TRPV1-/- mice have (1) an increase in the CB_1_ receptor coupling efficacy and (2) a shift from CB_1_ receptor-dependent LTD to LTP at the MPP–granule cell (GC) synapses.

We observed an increase in the CB_1_ receptor-related Gα_i1_, Gα_i2_, and Gαi3 subunits in synaptosomal fractions of TRPV1-/-. Furthermore, CB_1_ receptor functionality was altered in TRPV1-/- as the receptor showed a significant higher maximum coupling efficacy and a lower basal activation. These changes should be interpreted in the context of the decrease in CB_1_ receptors in the synaptosomal fractions (Egaña-Huguet et al., 2021). In fact, taking into account that [^35^S]GTPγS binding assays reflect a primary response of the system, the decrease in CB_1_ receptors could be expected to produce a decrease in maximal responses. However, the achievement of similar maximal responses in TRPV1-/- synaptosomal fractions together with a decrease in basal levels reveals an increase in the coupling efficacy induced by the agonist. Similarly, the decrease in the constitutive activation of G-proteins could be linked to the decrease in CB_1_ receptor expression. Actually, the CB_1_ receptor-induced suppression of the fEPSP revealed at the MPP–GC synapses (Peñasco et al., 2019) was not observed in TRPV1-/-. However, although CB_1_ receptors located on the glutamatergic synapses are tightly coupled to G protein signaling (Steindel et al., 2013), basal activation in the hippocampus could not only correspond to CB_1_ receptors as Gi/o proteins are also coupled to other metabotropic receptors besides the CB_1_ receptor (Conn and Pin, 1997). In addition, a drastic increase in the cannabinoid receptor-interacting protein 1a (CRIP1a) was detected previously in TRPV1-/- hippocampus (Egaña-Huguet et al., 2021). CRIP1a reduces the agonist-stimulated CB_1_ receptor internalization and attenuates CB_1_ receptor signaling, thus increasing neurotransmitter release (Booth et al., 2019; Oliver et al., 2020). Furthermore, CRIP1a overexpression in N18TG2 cells produces a robust stimulation of [^35^S]GTPγS binding to Gα_i1_ and Gα_i2_ subunits (Blume et al., 2015). Therefore, taking into account that TRPV1-/- courses with an increased Gα_i_ expression in the hippocampus, the potentiation observed in the [^35^S]GTPγS binding after CP 55,940 stimulation might be related to the CRIP1a rise (Blume et al., 2015; Booth et al., 2019; Oliver et al., 2020).

**Figure 5:**
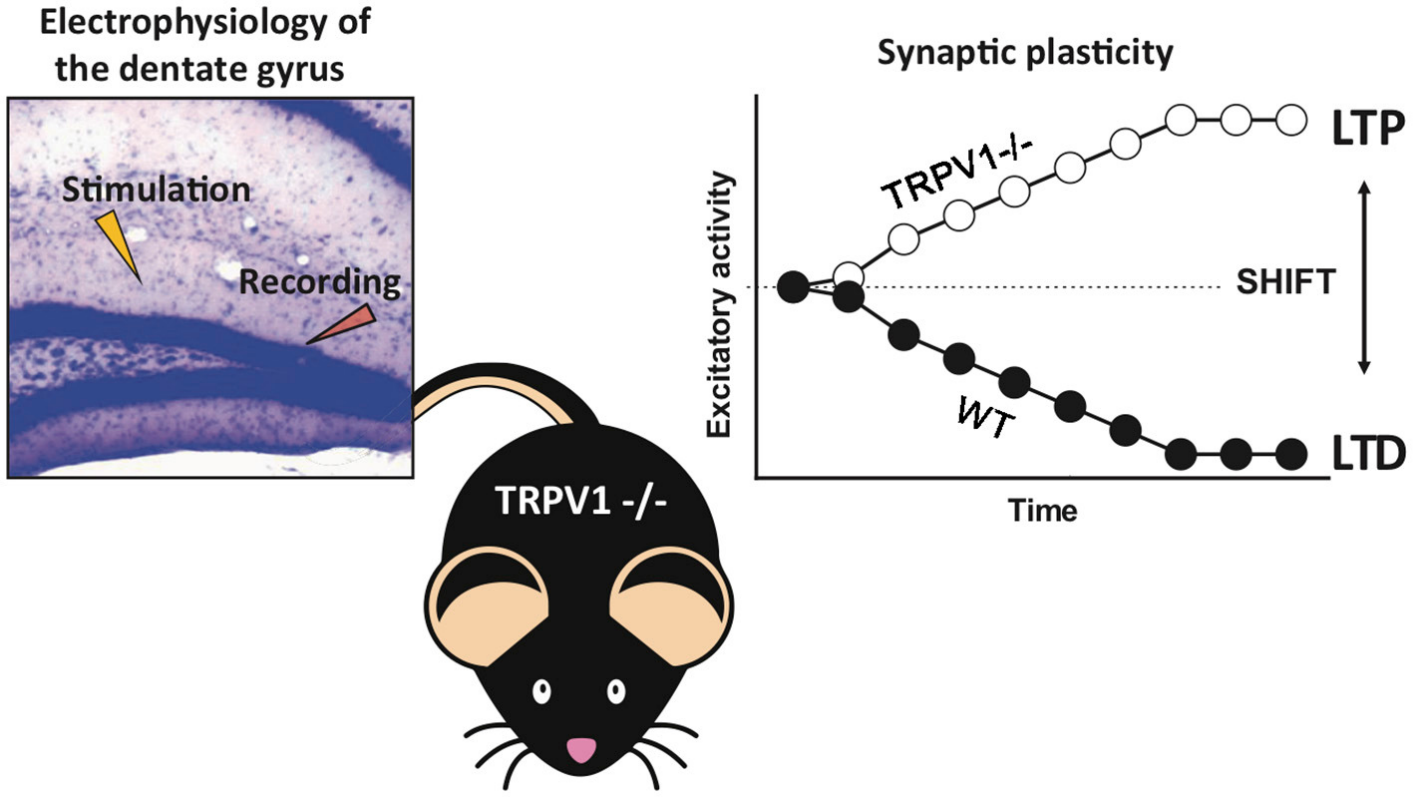
Summary of the main findings. The absence of TRPV1 shifts the cannabinoid CB_1_ receptor-dependent long-term depression to long-term potentiation at the excitatory medial perforant path–granule cell synapses in the mouse dentate molecular layer.

The present results also show a CB_1_ receptor-dependent shift to MPP-LTP in TRPV1-/- (Figure 5) after applying the LFS that elicits MPP-LTD under normal conditions (Peñasco et al., 2019; Fontaine et al., 2020). Interestingly, this type of potentiation is independent of NMDAR signaling, although eCB-eLTD requires NMDA receptor activation at other synapses (Sjöström et al., 2003; Bender et al., 2006; Lutzu and Castillo, 2021). These results highlight the importance of the crosstalk between CB_1_ and TRPV1 signaling in this form of synaptic plasticity.

The MPP-LTP in TRPV1-/- was abolished in the presence of DAGL or MAGL inhibitors. This suggests that 2-AG regulation may be a limiting factor for this kind of synaptic plasticity. Consequently, as observed for the eCB-eLTD at the MPP–GC synapses (Peñasco et al., 2019), CB_1_ receptor desensitization caused by high 2-AG concentration (Chanda et al., 2010; Schlosburg et al., 2010) could be responsible for the MPP-LTP blockade. Altogether, our findings suggest that 2-AG acting on CB_1_ receptors mediates MPP-LTD and MPP-LTP in WT and TRPV1-/-, respectively, in which levels and timing of 2-AG availability might be playing a role in this switch from LTD to LTP (Cui et al., 2018).

The MPP-LTP in TRPV1-/- might stand on the ability of LFS (1 Hz) of the MPP–GC synapses to induce CB_1_ receptor-independent LTD that relies on AEA, post-synaptic TRPV1, and AMPA receptor internalization (Chávez et al., 2010). However, MPP-LTP in TRPV1-/- was unaffected by the FAAH inhibitor URB597 (2 μM), indicating that AEA would not be involved. Actually, the conspicuous TRPV1 localization in the GC dendritic spines post-synaptic to the perforant path synaptic terminals in the outer two-thirds of the dentate ML (Puente et al., 2015) endorses TRPV1-mediated synaptic plasticity at the MPP–GC synapses. The differences observed between eCB-eLTD at the pre-synaptic (Peñasco et al., 2019; Fontaine et al., 2020) or post-synaptic sites of the MPP–GC synapses (Chávez et al., 2010) might be regarded as a synergistic effect of CB_1_ receptors reducing the glutamate release (Peñasco et al., 2019) and TRPV1 promoting AMPA receptor internalization (Chávez et al., 2010). Then, the change in CB_1_ receptors in the absence of TRPV1 (Egaña-Huguet et al., 2021) could be at the base of the shift from MPP-LTD to MPP-LTP. However, HFS (100 Hz) of the LPP–GC synapses actually triggers a CB_1_ receptor-mediated LTP that requires post-synaptic NMDA receptors and the production of mGluR5-dependent 2-AG (Wang et al., 2016). Furthermore, CB_1_ receptor activation at LPP synaptic terminals causes the assembly of LAT-sensitive actin filaments resulting in an increased release of glutamate (Wang et al., 2016, 2018). In our study, MPP-LTP in TRPV1-/- significantly decreased in the presence of LAT-A, suggesting a similar mechanism. Furthermore, our LFS triggered a similar LTP at the LPP–GC synapses in both WT (Wang et al., 2016, 2018) and TRPV1-/-, suggesting that the CB_1_ receptor-mediated LPP-LTP observed in our model is independent of TRPV1. Moreover, the MPP-LTP triggered in WT by pharmacological TRPV1 antagonism was significantly reduced by the CB_1_ receptor antagonist AM251 but it was unaffected by LAT-A, indicating that the MPP-LTP upon chemical TRPV1 blockade shares the CB_1_ receptor participation but not the intracellular signaling cascades turned on in the absence of TRPV1 at the MPP–GC synapses. In this sense, the NO donor SNAP blocked the MPP-LTP in TRPV1-/-. As TRPV1 is highly permeable to Ca^2+^ ions (Caterina et al., 1997; Caterina and Julius, 2001), it is plausible that reduced intracellular calcium caused by the absence of TRPV1 at the MPP–GC dendritic spine synapses would damp post-synaptic NO synthase and, therefore, decrease NO production needed to support pre-synaptic LTD in the hippocampus (Reyes-Harde et al., 1999). Under normal conditions, NO activates the pre-synaptic cGMP-dependent protein kinase (PKG) known to phosphorylate and inactivate the small GTPase RhoA (Sawada et al., 2001), as well as to regulate actin cytoskeleton (for review, Francis et al., 2010). Thus, the lack of NO in the absence of TRPV1 would eventually lead to the signaling of pre-synaptic molecular pathways that ultimately would enhance the release of glutamate (Wang et al., 2018) endorsing MPP-LTP. The possibility of indirect effects on MPP-LTP by CB_1_ receptors in other cells like astrocytes should also be considered, as astroglial CB_1_ receptors promote the excitatory LTD by favoring the local glutamate availability (Han et al., 2012) and endorse LTP at the distant excitatory synapses (Araque et al., 2017).

Previous studies have shown that TRPV1-/- mice exhibit learning and conditioned fear deficits as well as anxiety-like behaviors, which were related to a decrease in excitatory LTP at CA1 synapses (Marsch et al., 2007). Also, the disappearance of CB_1_ receptor-dependent LTD at the MPP synapses in the adult brain after exposure to intermittent ethanol intake during the adolescence and the associated recognition memory deficits were rescued by increasing 2-AG (Peñasco et al., 2020). So the shift from CB_1_ receptor-dependent MPP-LTD to MPP-LTP in TRPV1-/- mice might be affecting memory as the hippocampus in general (Eichenbaum et al., 2012), and the MPP synapses, in particular, are involved in spatial memory processing (Fyhn et al., 2004; Hargreaves et al., 2005).

Altogether, the biochemical and anatomical changes taking place in the endocannabinoid system of TRPV1-/- mice (Egaña-Huguet et al., 2021), together with the increase in Gα_i1_, Gα_i2_, and Gα_i3_ proteins, the low basal CB_1_ receptor activation, the high CB_1_ receptor coupling efficacy, and the shift from MPP-LTD to MPP-LTP demonstrated in this study support a functional crosstalk between TRPV1 and CB_1_ receptors in the dentate gyrus (Figure 5).

## Data Availability Statement

The original contributions presented in the study are included in the article/Supplementary Material, further inquiries can be directed to the corresponding author.

## Ethics Statement

The animal study was reviewed and approved by Committee of Ethics for Animal Welfare of the University of the Basque Country (CEEA/M20/2015/105; CEIAB/M30/2015/106).

## Author Contributions

JE-H: conceptualization, methodology, formal analysis, data curation, visualization, validation, investigation, and writing original draft. MS-E: methodology, formal analysis, investigation, visualization, validation, and writing original draft. SA and IB-D: methodology, visualization, and investigation. ES-G: writing original draft. GG: methodology, investigation, data curation, and writing-review and editing. SB: methodology, investigation, and data curation. JS: funding acquisition and writing-review and editing. IG: resources and investigation. NP and IE: conceptualization, methodology, formal analysis, and data curation. PG: conceptualization, supervision, project administration, funding acquisition, and writing-review and editing. All authors contributed to the article and approved the submitted version.

## Conflict of Interest

The authors declare that the research was conducted in the absence of any commercial or financial relationships that could be construed as a potential conflict of interest.

## Abbreviations

ACSF: Artificial cerebrospinal fluid
AEA: Arachidonoyl-ethanolamine or anandamide
AMPA: _D_-Amino-3-hydroxy-5-methyl-4-isoxazole-propionic acid
BSA: Bovine serum albumin
CA1: Region 1 of Cornu ammonis
CB_1_ receptor: Type I cannabinoid receptor
CRIP1a: Cannabinoid receptor-associated protein 1a
DAGL: Diacylglycerol lipase
DMSO: Dimethyl sulfoxide
eCB: Endocannabinoid
ECS: Endocannabinoid system
eCB-eLTD: Endocannabinoid-mediated long-term depression of excitation
eLTD: Long-term depression of excitation
FAAH: Fatty acid amide hydrolase
fEPSP: Field excitatory post-synaptic potential
FIJI: Fiji is just IMAGE J
LFS: Low-frequency stimulation
LPP: Lateral perforant path
LTD: Long-term depression
LTP: Long-term potentiation
MAGL: Monoacylglycerol lipase
mGluR: Metabotropic glutamate receptors
mGluR5: Metabotropic glutamate receptor 5
ML: Molecular layer
MPP: Medial perforant pathway
NMDAR: *N*-methyl-_D_-aspartate receptor
PBS: Phosphate-buffered saline
PP: Perforant path
PTX: Picrotoxin
PVDF: Polyvinylidene difluoride
RT: Room temperature
SDS: Sodium dodecyl sulfate
S.E.M.: Standard error of the mean
TRP: Transient receptor potential
TRPV1: Transient potential receptors of vanilloid type 1
TRPV1-/-: TRPV1 knockout
WT: Wild type
2-AG: 2-Arachidonoyl glycerol
[^35^S]GTPγS: Guanosine-5′-O-(3-[^35^S]thiotriphosphate).
