# Supplementary material for: Lack of the Transient Receptor Potential Vanilloid 1 Shifts Cannabinoid-Dependent Excitatory Synaptic Plasticity in the Dentate Gyrus of the Mouse Brain Hippocampus
Source: Front Neuroanat. 2021 Jul 7;15:701573. doi: 10.3389/fnana.2021.701573 (PMC8294191; doi:10.3389/fnana.2021.701573)
Supplement: Supplementary file 1 [file Data_Sheet_1.PDF]

Supplementary material

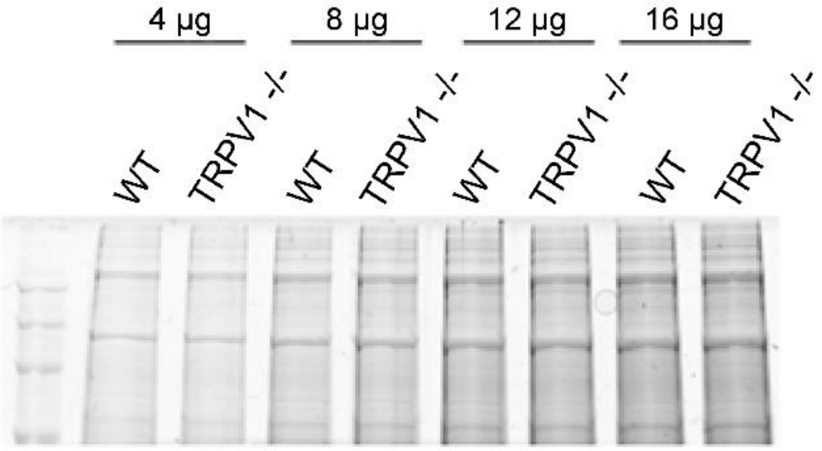

Figure S1. Representative image of Coomassie blue-stained SDS-PAGE with increasing amounts of hippocampal synaptosomes of wild-type (WT) and TRPV1 knockout (TRPV1-/-) mice. The top of polyacrylamide gel, containing high molecular weight proteins, was cut and stained with a Coomassie blue dye as a loading control. The rest of the gel, where proteins of interest migrate, is transferred to PVDF membranes to proceed with the immunoblot.

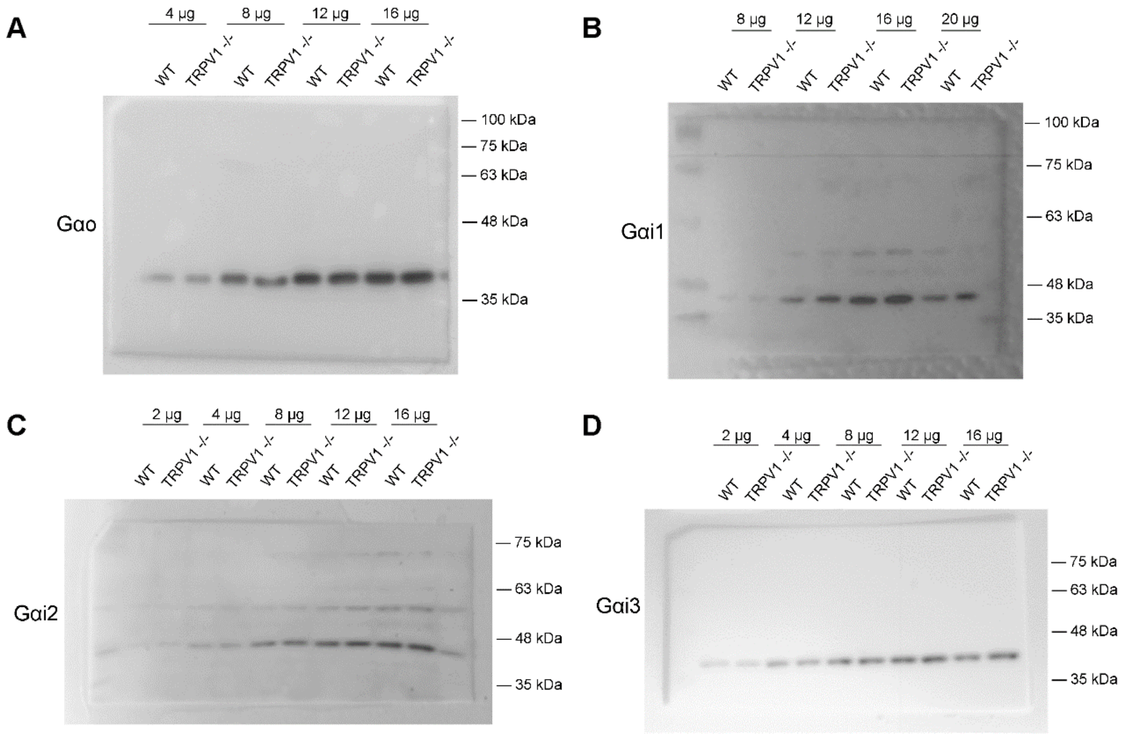

Figure S2. Untruncated image of Western blots carried out by immunoblotting increasing amounts of hippocampal synaptosomes of wild-type (WT) and TRPV1 knockout (TRPV1-/-) mice. For representation, colorimetric and chemiluminiscence images were merged by using Image-J. The molecular weights depicted correspond to the signal of the standard markers. The protein loading was checked by Coomassie Brilliant Blue gel

staining method. **A)** Immunoblot for $G\alpha_o$ protein. **B)** Immunoblot for $G\alpha i_1$ protein. **C)** Immunoblot for $G\alpha i_2$ protein. **D)** Immunoblot for $G\alpha i_3$ protein.

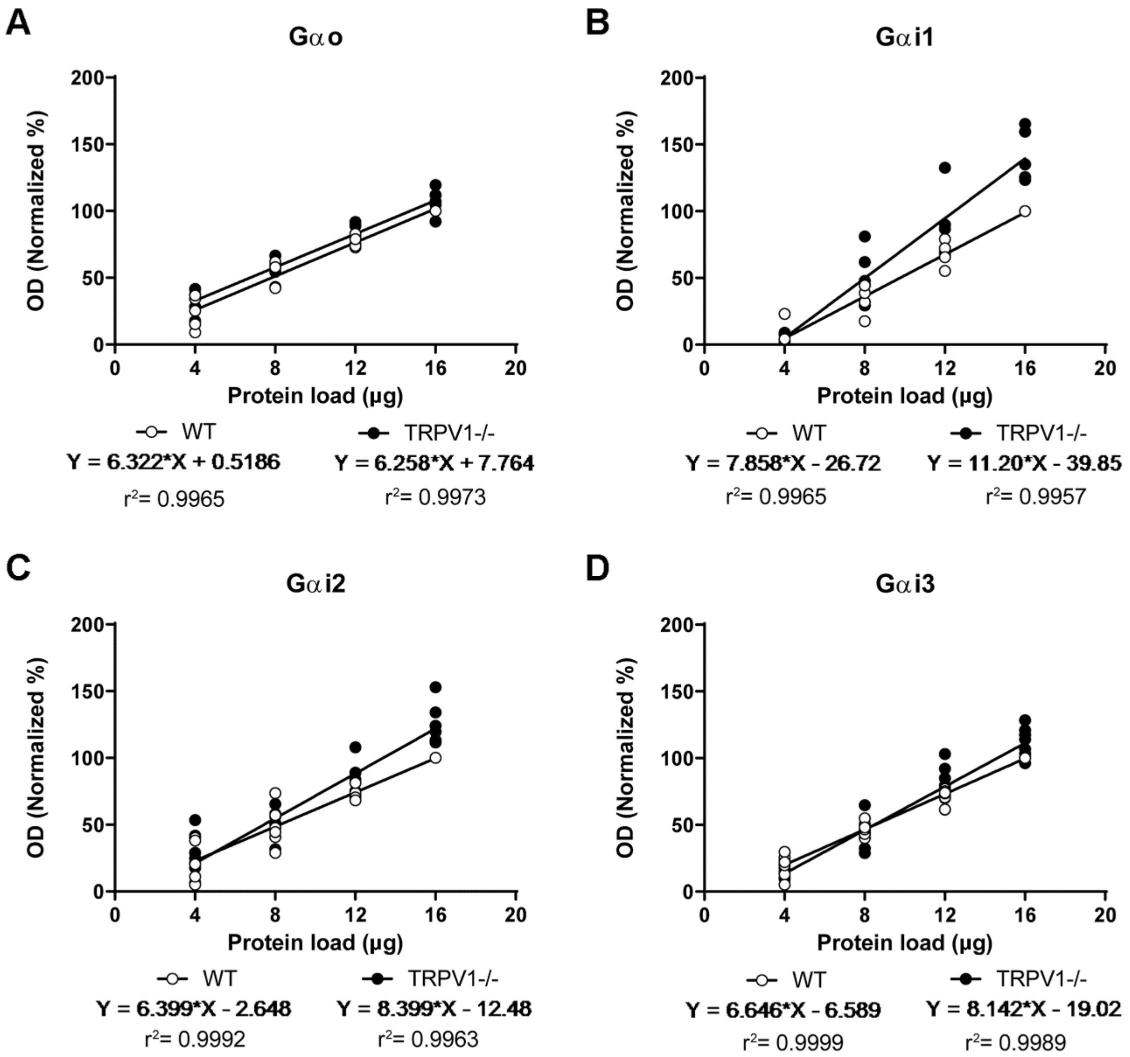

Figure S3. Regression analysis of curves generated by optical density (OD) values of the immunoreactive signals of Gαi/o proteins in hippocampal synaptosomes of wild-type (WT) and TRPV1 knockout (TRPV1-/-) mice. Linear regressions curves for: **A)** $G\alpha_o$ (n=7); **B)** $G\alpha i_1$ (n=7); **C)** $G\alpha i_2$ (n=7) and **D)** $G\alpha i_3$ (n=7). Fisher's exact test. $p > 0.05$; $^{**}p < 0.01$.

Individual integrated optical density (OD) values derived from immmunoreactive signals from each protein loading were normalized against the immunoreactive signal obtained from the maximal loading of the WT mice. Plotting the increasing amounts of protein and integrated optical densities (OD), allowed us to obtain a linear regression equation for each sample. The ratio between the slopes of the curves allowed us to determinate the fold of change on the expression of the analyzed protein.
